# High transcriptome plasticity drives phosphate starvation responses in tomato

**DOI:** 10.1007/s44154-022-00035-4

**Published:** 2022-03-18

**Authors:** Viswanathan Satheesh, Jieqiong Zhang, Jinkai Li, Qiuye You, Panfeng Zhao, Peng Wang, Mingguang Lei

**Affiliations:** 1 Shanghai Center for Plant Stress Biology, CAS Center for Excellence in Molecular Plant Sciences, Chinese Academy of Sciences, Shanghai, 200032 China; 2 School of Life Science and Technology, Tongji University, Shanghai, 200092 China; 3 University of Chinese Academy of Sciences, Beijing, 100049 China

**Keywords:** Tomato, Pi starvation, Transcriptome, Gene expression, Alternative splicing, Co-expression network

## Abstract

Tomato is an important vegetable crop and fluctuating available soil phosphate (Pi) level elicits several morpho-physiological responses driven by underlying molecular responses. Therefore, understanding these molecular responses at the gene and isoform levels has become critical in the quest for developing crops with improved Pi use efficiency. A quantitative time-series RNA-seq analysis was performed to decipher the global transcriptomic changes that accompany Pi starvation in tomato. Apart from changes in the expression levels of genes, there were also alterations in the expression of alternatively-spliced transcripts. Physiological responses such as anthocyanin accumulation, reactive oxygen species generation and cell death are obvious 7 days after Pi deprivation accompanied with the maximum amount of transcriptional change in the genome making it an important stage for in-depth study while studying Pi stress responses (PSR). Our study demonstrates that transcriptomic changes under Pi deficiency are dynamic and complex in tomato. Overall, our study dwells on the dynamism of the transcriptome in eliciting a response to adapt to low Pi stress and lays it bare. Findings from this study will prove to be an invaluable resource for researchers using tomato as a model for understanding nutrient deficiency.

**Supplementary Information:**

The online version contains supplementary material available at 10.1007/s44154-022-00035-4.

## Introduction

Availability of phosphorus (P), an essential macronutrient, is one of the major limiting factors in crop productivity. Soil P exists in both the inorganic (Pi) and organic forms, and conversion to the Pi form is essential for absorption by plants (Horst et al., 2001; Hinsinger, 2001). Unfortunately, farmers have adopted indiscriminate use of chemical fertilizers to manage low Pi (0.1–10 μm; Hinsinger, 2001) in agricultural ecosystems to enhance crop growth and productivity. Plants have evolved adaptive mechanisms, including morphological and biochemical responses, to adapt to low Pi stress. These mechanisms exert local and systemic control over Pi nutrition. Under Pi-depleted condition, primary root growth inhibition, increased root hair density, and enhanced lateral root growth are important root system architectural (RSA) changes (López-Bucio et al., 2003; Desnos, 2008). The RSA changes are significant as they maximize Pi acquisition (Lynch and Brown 1998). Increased expression of Pi transporters, induction and secretion of acid phosphatase and organic acid, and accumulation of anthocyanin and starch are other Pi starvation responses (PSRs) (Raghothama, 1999; López-Bucio et al., 2002; Yuan and Liu 2008). To develop low Pi-tolerant crops, a strategy for addressing the problem, understanding the molecular mechanisms driving these morphological and biochemical responses is essential.

There has been significant progress in the study of PSRs in model plants, predominantly in dicot Arabidopsis and monocot rice. PHR1 (OsPHR2 in rice), a Myb-like transcription factor, plays a central role in PSR (Rubio et al. 2001; Bustos et al. 2010). PHR1 binds to an imperfect palindromic sequence (GNATAT), found in the promoter regions of many Pi starvation-induced (PSI) genes, and induces their expression (Rubio et al. 2001). The SPX domain-containing protein, SPX1 (and its rice homologs OsSPX1/2), acts as a sensor binding to the inositol pyrophosphate InsP_8_ under Pi-replete condition to further bind to PHR1 preventing it from binding to the PSI genes (Puga et al. 2014; Wang et al. 2014; Wild et al. 2016; Qi et al. 2017; Dong et al. 2019; Zhu et al. 2019; Ried et al. 2021; Zhou et al. 2021). Under Pi-depleted condition, reduced InsP_8_ level releases PHR1 from the SPX1-InsP8-PHR1 complex and PHR1 activates the expression of PSI genes, including Pi transporters, acid phosphatases, and other transcriptions factors (Chiou and Lin, 2011; Dong et al. 2019; Zhu et al. 2019).

Although great strides have been made in Arabidopsis and rice, the Pi-sensing and signaling mechanism in other plants is still poorly understood (Wu et al. 2013; Lopez-Arredondo et al. 2014). In this context, studying gene expression profiles has become commonplace with the advent of next-generation sequencing (NGS) technologies. Several studies have reported the transcriptional changes under Pi starvation, and they have shed more light on the transcriptome landscape of the plants providing valuable insights into the mechanisms underlying PSR (Misson et al. 2005; Bustos et al. 2010; Secco et al. 2013; Dong et al. 2018; Tian et al. 2021). The first large-scale transcriptome study was performed in Arabidopsis using the microarray technology (Misson et al. 2005). Recent studies have used the power of NGS technology in rice (Secco et al. 2013; Dong et al. 2018) and tomato (Tian et al. 2021) adding a wealth of information to the short- and long-term changes that occur in Arabidopsis, rice and tomato during Pi starvation. Alternative splicing (AS) is a significant phenomenon that brings about the variety in the number of proteins that an organism might code for with relatively fewer protein-coding genes. AS is widespread and can be regulated in a tissue- or condition-specific manner (Thatcher et al. 2016). While there is very little overlap between differentially expressed genes (DEGs) and differentially alternatively spliced genes (DASGs) under stress, DASGs can elicit stress-specific responses (Li et al. 2013; Calixto et al. 2018; Dong et al. 2018; Liu et al. 2018; Tian et al. 2021). Therefore, the study of both DEGs and DASGs has gained significant importance in the context of stress response.

Tomato, a well-characterized model crop for basic research, has an annual production of 100 million tons (FAO, 2015) worldwide, making it an important vegetable crop. Unlike Arabidopsis and rice, research on Pi starvation responses in tomato is limited (Liu et al. 1997; Baldwin et al. 2001; Wang et al. 2002; Baldwin et al. 2008; Muneer and Jeong 2015; Suen et al. 2015), but recent publications suggest a change in focus (Zhao et al. 2019; Zhang et al. 2019; Pfaff et al. 2020; Srivastava et al. 2020; Tian et al. 2021; Zhang et al. 2021). In addition to anthocyanin accumulation, tomato also produces reactive oxygen species (ROS) under Pi starvation (Muneer and Jeong 2015). In maize, tolerance to low Pi has been attributed to the effective scavenging of ROS (Du et al. 2016). Under stress, ROS leads to cell death, a process that, in its active form, is essential for the growth and development of plants. Necrosis is a passive form wherein cell death occurs in plants under stress due to the accumulation of molecules such as ROS. The process is indiscriminate and often irreversible. Genome-wide expression analysis in tomato under Pi starvation showed that several genes were differentially expressed along with a significant number of alternatively spliced transcripts some of which are PSR genes (Tian et al. 2021).

In this study, with the help of high-throughput sequencing, we present the transcriptomic landscape of root and shoot of tomato seedlings under Pi-depleted condition. The data generated in this study provides an overview of the complexities and the plasticity of the tomato transcriptome to maintain Pi-homeostasis during the early stages of growth. We have studied changes in transcript abundances both at the gene and isoform levels to reveal the intricate changes accompanying low Pi stress. This study will serve as a significant resource for researchers for understanding the low Pi-driven molecular responses in tomato.

## Results

### Physiological and molecular changes in seedlings under Pi deficiency

**Fig. 1:**
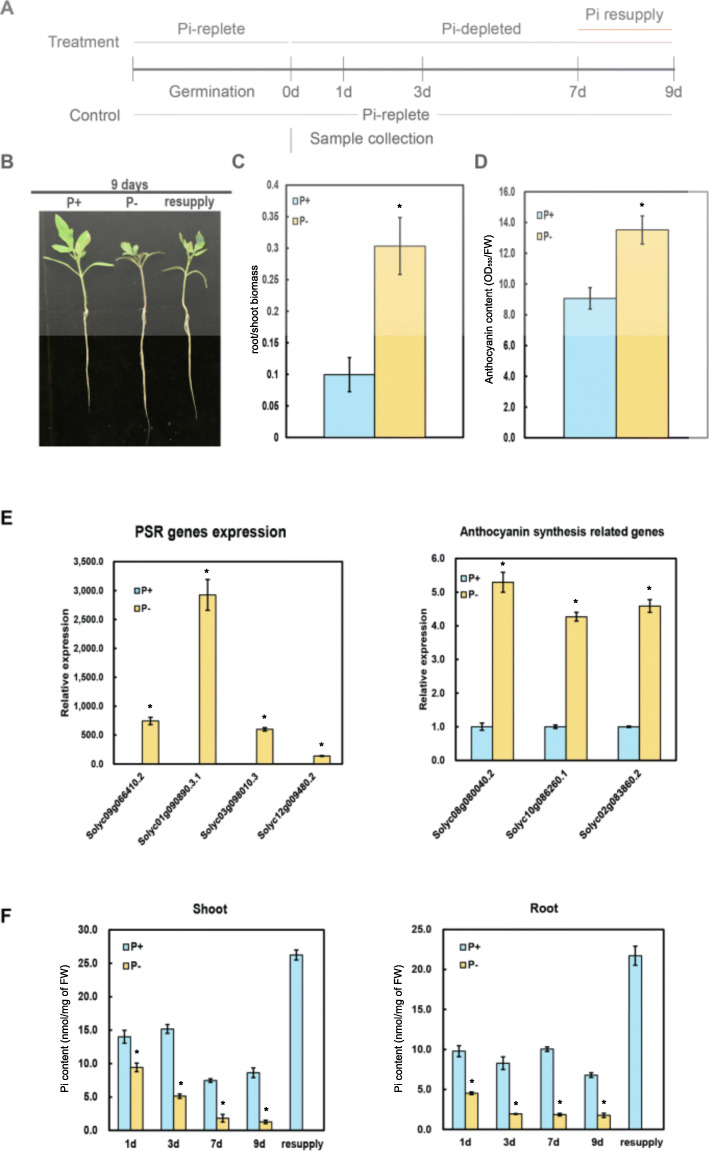
Phenotypic and physio-molecular changes in tomato under Pi starvation. **A**. The time series experiment schematic. 0 d, 1 d, 3 d, 7 d, and 9 d refer to time in days after Pi starvation. Seedlings are Pi-starved 6 days after germination up to 7 d and resupplied with Pi for a further 2 d. **B**. Phenotype of the seedlings at 9d for seedlings under P-replete (P+), Pi-depleted (P-), and Pi-resupply (resupply). The root:shoot biomass ratio (**C**), anthocyanin content (**D**), gene expression levels of PSR genes and anthocyanin biosynthesis genes (**E**), and Pi content in shoot and root (**F**) after 7 d of Pi starvation. Error bars are mean ± SD. All experiments were performed at least twice with three biological replicates. The asterisk represents statistically significant difference at *p* < 0.05

To understand how changes in the availability of Pi affect tomato and to define the transcriptome under low Pi condition, a time-course experiment was conducted to identify important stages in the progression of Pi starvation in tomato during the seedling stage. Five-day(d)-old seedlings were grown in hydroponic medium with (P+) or without (P-) supplemental Pi and grown for 1, 3, 7 and 9 d (Fig. 1A). For Pi resupply, seedlings grown in P- medium for 7 d were transferred to P+ medium for 2 more d. The seedling phenotype at 9 d were observed for Pi-replete, Pi-depleted and Pi-resupply conditions (Fig. 1B). As expected, Pi deficiency resulted in a significant increase in root:shoot biomass ratio, anthocyanin content, and the expression of PSR and anthocyanin biosynthetic genes at 7 d (Fig. 1C-E). Pi content both in the shoot and root was dramatically decreased (Fig. 1F). Pi resupply greatly increased Pi content in Pi-starved seedling, even much higher than that grown in P+ medium (Fig. 1F).

**Fig. 2:**
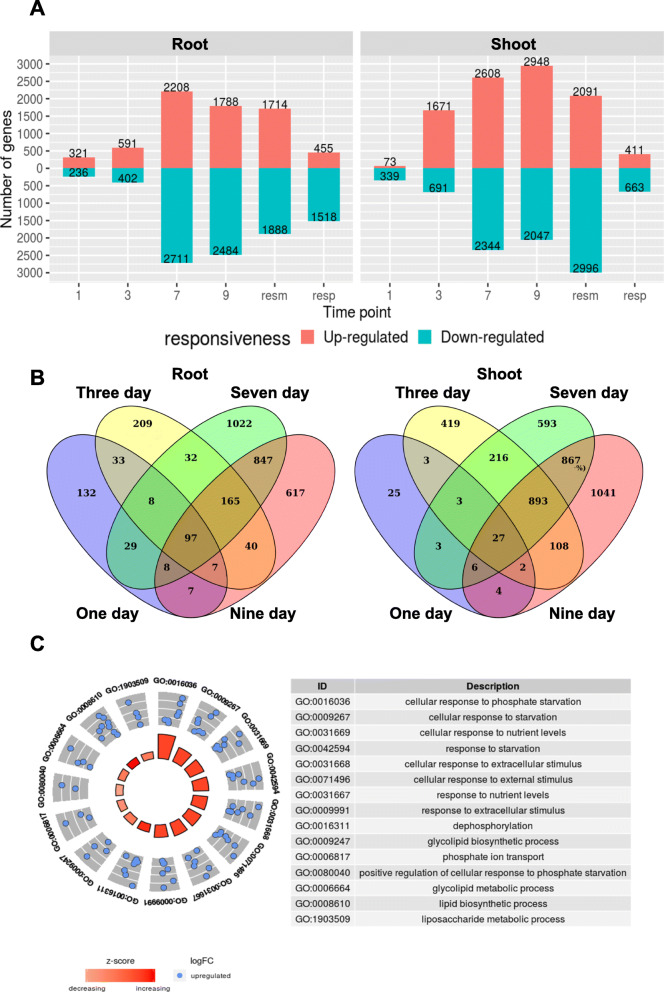
Major transcriptomic changes occur 7 d after Pi starvation in tomato seedlings. **A**. Differential gene expression levels in root and shoot tissues. **B**. Overlap of upregulated genes in all four timepoints in root and shoot samples. **C**. GO enrichment analysis of the 97 upregulated genes overlapping in all four timepoints in the root samples showing enrichment for Pi starvation responsiveness

Root and shoot samples were collected at these time points with three biological replicates per treatment. Total RNA was isolated from 54 samples for transcriptome sequencing, which yielded ~ 1.34 billion clean 150 bp paired-end reads. These clean reads were mapped to the tomato genome build SL2.50 and its corresponding annotation, ITAG2.4. On an average, ~ 90% of the reads mapped uniquely to the genome under different treatment conditions and different tissues (Table 1). Differential expression analysis was performed using DESeq2. Genes that showed a two-fold difference with a false discovery rate less than 0.05 were considered differentially expressed (Fig. 2A; Supplementary Data 1). All the genes that showed differential expression of more than 10,000-fold were discarded to control for false positives (Rawat et al. 2015). While 557/412 (root/shoot) genes were differentially expressed one DAT, only 73 genes were upregulated in the shoot as opposed to 321 in root. However, there was a dramatic increase in the number of differentially expressed genes (DEGs) in shoot after 3 d (2362) when compared with root (993). Most of the enriched terms for the genes that are upregulated 1 DAT in root are “negative regulation of enzymatic activity” and a set of genes that are specific to phosphate starvation (FDR < 0.05). As the duration of the treatment extends into the third day, the molecular response becomes more specific with the enrichment of such terms as “cellular response to nutrient levels/starvation/extracellular stimulus/external stimulus/phosphate starvation”. The most significant changes in expression were observed at 7/9 d in both root (4919/4272 DEGs) and shoot (4952/4995 DEGs). A list of top 40 genes that are upregulated in root or shoot tissues is given in Table 2. To obtain a better picture of the expression profile of the various genes under Pi starvation across the different time points and tissues, a weighted gene co-expression network analysis was performed. An overlap analysis showed that 97 genes commonly upregulated from the first day of Pi starvation onwards (Fig. 2B). GO enrichment analysis of these genes showed that the terms for “Pi starvation response” were enriched (Fig. 2C).

**Table 1:** RNAseq reads mapped to the tomato genome

	P+		P-		Resupply		Total
	Root	Shoot	Root	Shoot	Root	Shoot	
Percentage of reads uniquely mapped	88.56	88.69	90.32	92.03	91.03	93.5	90.16
Total reads	315,213,219	275,795,595	319,883,111	285,717,224	71,815,642	75,615,975	1,344,040,766
Number of splices	260,797,139	236,110,793	279,316,876	253,666,454	61,291,538	68,549,800	1,159,732,600
Number of non-canonical splices	492,038	401,804	465,521	378,577	104,710	112,086	1,954,736

**Table 2:** Top 40 upregulated genes 7 DAT in root and shoot

Gene ID	Description	Log2Fold
**Root**		
Solyc01g005940.2	phytoene synthase 2	7.22
Solyc05g008910.2	PREDICTED: uncharacterized protein LOC101245357 isoform X1	7.04
Solyc05g014550.1	TLC domain-containing protein At5g14285-like	7.03
Solyc12g009480.1	SPX domain-containing protein 1	7.42
Solyc07g053900.2	PREDICTED: uncharacterized protein LOC101245965	8.51
Solyc04g080480.1	long-chain-fatty-acid--AMP ligase FadD26-like	10.23
Solyc01g100020.2	phospholipase D zeta 1-like	7.65
Solyc03g098010.2	purple acid phosphatase 17-like	8.34
Solyc01g091870.2	SPX domain-containing membrane protein	7.59
Solyc08g079300.2	geraniol 8-hydroxylase-like	7.79
Solyc06g072960.1	ABC transporter B family member 19-like	7.13
Solyc08g060920.2	SPX domain-containing protein 1-like	6.95
Solyc12g006130.1	F-box protein At3g07870-like	9.91
Solyc01g090890.2	SPX domain-containing protein 3	8.98
Solyc01g059900.2	dirigent protein 11-like	7.12
Solyc06g062540.2	putative phosphatase	7.02
Solyc03g078500.2	7-deoxyloganetin glucosyltransferase-like	9.11
Solyc01g091760.2	ethylene-responsive transcription factor ERF010-like	7.17
Solyc11g005610.1	nodulation-signaling pathway 2 protein-like	9.76
Solyc08g066720.1	carotenoid 9 10 (9′ 10′)-cleavage dioxygenase 1-like	13.10
Solyc04g080920.1	purple acid phosphatase 2-like	11.16
Solyc02g092060.1	3-oxoacyl-[acyl-carrier-protein] reductase FabG-like	11.11
Solyc07g042400.2	PREDICTED: uncharacterized protein LOC101244203	7.82
Solyc03g031410.1	Unknown protein	8.64
Solyc10g080870.2	alkane hydroxylase MAH1-like	7.26
Solyc12g006720.1	Unknown protein	8.14
Solyc07g055550.1	cytochrome P450 CYP72A219-like	10.62
Solyc06g072950.1	ABC transporter B family member 19-like	12.67
Solyc04g074540.2	alcohol dehydrogenase-like 1	7.58
Solyc08g081620.2	endo-14-beta-glucanase precursor	7.22
Solyc01g006640.1	4-coumarate--coa ligase-like 1	8.38
Solyc01g100910.2	WAT1-related protein At1g09380-like	6.90
Solyc04g015110.2	WEB family protein At1g12150-like	9.83
Solyc12g062340.1	Unknown protein	8.16
Solyc02g089450.1	protein GLUTAMINE DUMPER 3-like	7.65
Solyc10g005390.2	(3S6E)-nerolidol synthase 1-like	9.98
Solyc12g014580.1	major pollen allergen Ole e 6-like	7.76
Solyc09g009560.1	CEN-like protein 2	7.04
Solyc04g056450.2	PREDICTED: uncharacterized protein LOC107015997	7.35
Solyc12g036130.1	ABC transporter C family member 12-like isoform X5	6.97
**Shoot**		
Solyc04g015120.2	U-box domain-containing protein 36	9.15
Solyc06g062560.1	putative phosphatase	12.05
Solyc09g091910.1	purple acid phosphatase 15 isoform X1	8.10
Solyc06g062540.2	putative phosphatase	10.62
Solyc09g066410.1	inorganic phosphate transporter 1–4-like	9.74
Solyc06g062550.2	putative phosphatase	10.95
Solyc01g090890.2	SPX domain-containing protein 3	10.52
Solyc03g078500.2	7-deoxyloganetin glucosyltransferase-like	11.43
Solyc12g006130.1	F-box protein At3g07870-like	11.13
Solyc04g080920.1	purple acid phosphatase 2-like	9.12
Solyc11g072800.1	putative respiratory burst oxidase homolog protein H isoform X1	10.33
Solyc02g067190.2	casparian strip membrane protein 1	9.68
Solyc09g061730.1	PREDICTED: uncharacterized protein LOC101261061	9.40
Solyc02g089450.1	protein GLUTAMINE DUMPER 3-like	10.15
Solyc07g042400.2	PREDICTED: uncharacterized protein LOC101244203	8.12
Solyc12g062340.1	Unknown protein	9.69
Solyc10g081890.1	aluminum-activated malate transporter 8-like	9.05
Solyc03g032220.2	probable caffeoyl-CoA O-methyltransferase At4g26220	8.66
Solyc01g096140.2	aluminum-activated malate transporter 10	8.73
Solyc10g083250.1	casparian strip membrane protein 1	7.91
Solyc05g050880.2	cationic peroxidase 1-like	8.80
Solyc03g031410.1	Unknown protein	8.85
Solyc04g015110.2	WEB family protein At1g12150-like	11.14
Solyc08g008200.1	cation/H(+) antiporter 18-like	8.92
Solyc10g080270.1	hypothetical protein A4A49_23646	7.96
Solyc10g049720.1	transcription factor bHLH139-like	8.00
Solyc09g074500.1	Unknown protein	8.92
Solyc12g062940.1	probable glycosyltransferase At5g03795	8.05
Solyc09g098080.2	anthocyanidin 3-O-glucosyltransferase 2-like	8.08
Solyc09g063150.2	glutathione transferase GST 23-like	7.98
Solyc04g008670.1	gibberellin 2-beta-dioxygenase 8-like	8.89
Solyc04g008250.1	purple acid phosphatase 8-like	10.22
Solyc05g053150.1	transcription factor MYB24-like	10.39
Solyc06g072950.1	ABC transporter B family member 19-like	8.94
Solyc01g018020.1	transketolase	8.45
Solyc08g005940.1	trypsin proteinase inhibitor precursor	9.23
Solyc10g006340.2	serine/threonine-protein kinase CDL1-like	8.58
Solyc02g086310.1	non-specific lipid-transfer protein 2-like	9.32
Solyc02g086400.1	DNA mismatch repair protein MLH3 isoform X1	8.28
Solyc08g062200.1	PREDICTED: uncharacterized protein LOC104648819	10.97

### Weighted gene co-expression network analysis

**Fig. 3:**
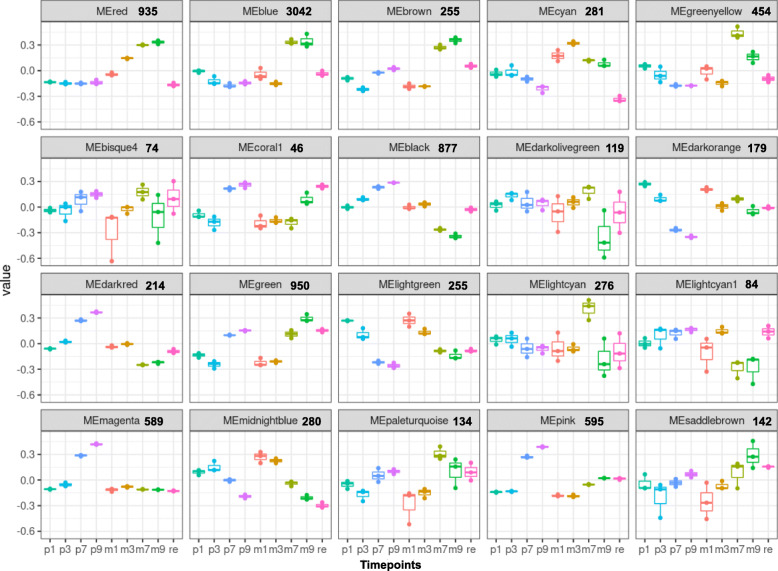
Weighted gene coexpression network analysis of root samples. Twenty significant modules obtained from the WGCNA analysis of the root samples are shown. ME – Module Eigengene; the numbers beside the module name represent the number of genes in the respective module. p1-p3-p7-p9: samples under Pi-replete condition at 1, 3, 7, and 9 days after starvation. m1-m3-m7-m9: samples under Pi-depleted condition at 1, 3, 7, and 9 days after starvation. re: Pi resupply samples

**Fig. 4:**
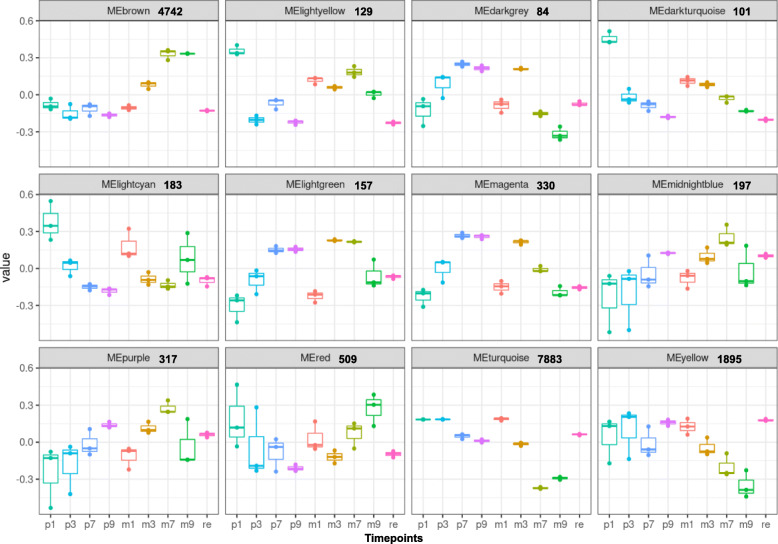
Weighted gene coexpression network analysis of shoot samples. Twelve significant modules obtained from the WGCNA analysis of the shoot samples are shown. ME – Module Eigengene; the numbers beside the module name represent the number of genes in the respective module. p1-p3-p7-p9: samples under Pi-replete condition at 1, 3, 7, and 9 days after starvation. m1-m3-m7-m9: samples under Pi-depleted condition at 1, 3, 7, and 9 days after starvation. re: Pi resupply samples

**Fig. 5:**
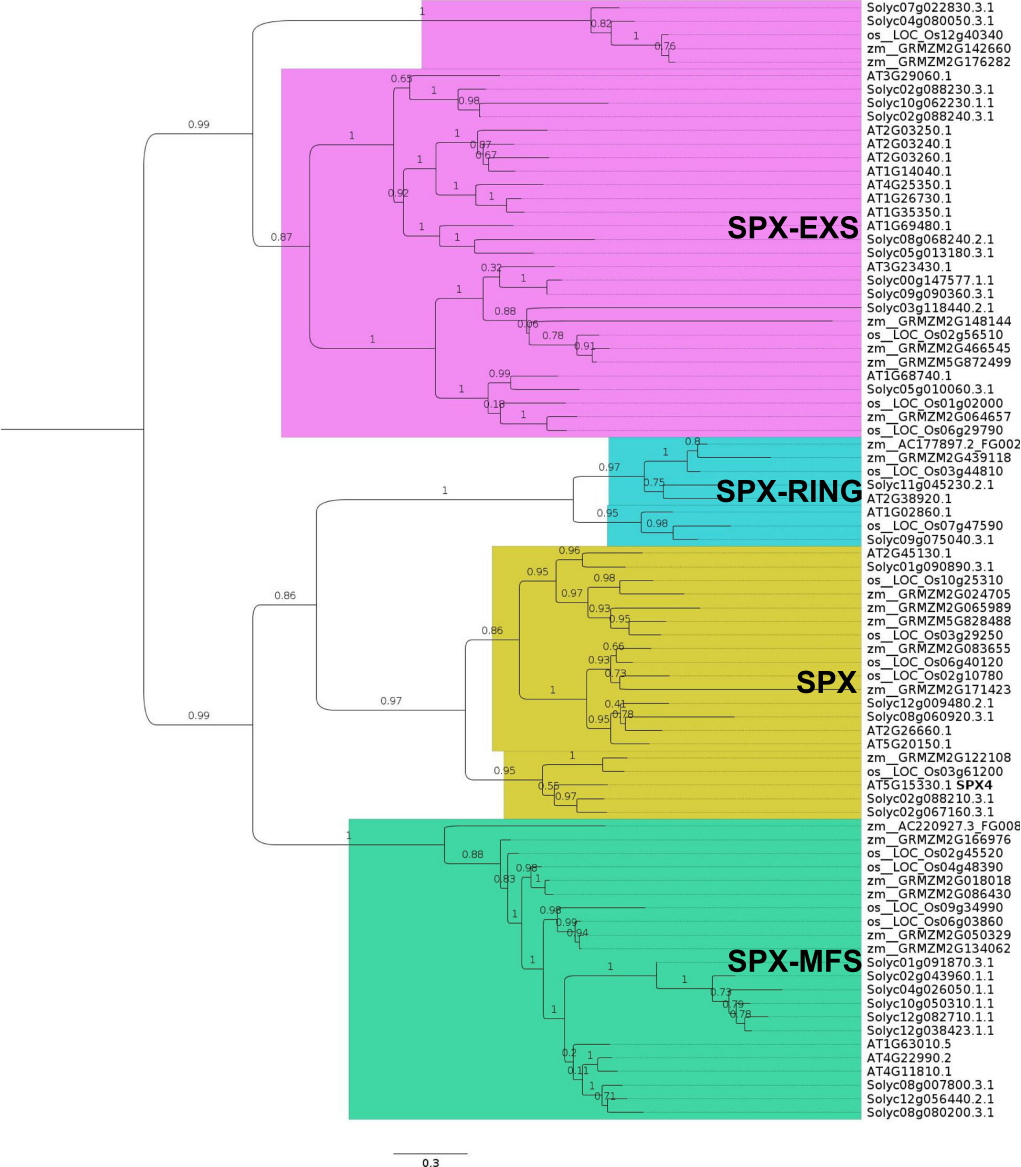
Phylogenetic analysis of SPX domain proteins from tomato, Arabidopsis, rice and maize. All SPX domain-containing proteins were identified from tomato, Arabidopsis, rice and maize genome and a phylogenetic tree was generated. The phylogeny reveals the four different classes of SPX proteins viz., SPX domain only, SPX-EXS domain, SPX-RING, and SPX-MFS domain proteins. There are five SPX domain only proteins in tomato

The R package WGCNA (weighted gene co-expression network analysis) was used to find modules comprising genes of high correlation in root and shoot samples separately. For this analysis, all expressed genes from all samples were taken for the analysis. A Pearson correlation matrix was used to create a signed network with the TOMsimilarityFromExpr function. A signed network was created to ensure only positive correlations were taken into consideration. A soft threshold power of 7 was used as it resulted in a scale-free topology (R^2^ value of 0.8). If the scale-free topology fit index fails to reach values above 0.8 for reasonable powers (less than 15 for unsigned or signed hybrid networks, and less than 30 for signed networks) and the mean connectivity remains relatively high (in the hundreds or above), chances are the data exhibit a string driver that makes a subset of the samples globally different from the rest. The difference causes high correlation among large groups of genes which invalidates the assumption of the scale-free topology approximation. A higher scale-free topology of R^2^ greater than 0.9 was not achieved, and therefore 0.8 was set as the score for determining it. Co-expressed genes identified by this analysis are presented as modules, which can be further analyzed. For the analysis, we used all genes that had a total of 10 counts in all samples put together (26,350 genes). In the root samples, the genes clustered into 65 different modules of which 20 are shown in Fig. 3. A similar approach was taken for understanding the expression profiles in shoot samples by creating signed networks (Fig. 4). In the root samples, the red module consisting of 935 genes, showed that the genes are all activated on Pi-depletion and their expression continues to increase up to 9 d. All genes are downregulated to their basal levels after resupply. This indicates that this set of genes are the Pi-specific inducible genes that are activated early on during the stress. Seven SPX domain containing genes are found in this module. Solyc01g090890 (SPX3), Solyc01g091870 (PHT5;3), Solyc02g067160 (SPX4-like), Solyc02g088210 (SPX4-like), Solyc05g010060 (PHO1;H1), Solyc08g060920 (SPX1) and Solyc12g009480 (SPX2). Except for Solyc01g091870 and Solyc05g010060—SPX-MFS and SPX-EXS types, respectively—the other five are SPX domain only genes (Fig. 5, 6A). Therefore, it is interesting that all five SPX domain only containing genes are found in the red module, emphasising the importance of these genes in Pi response, probably in sensing given that the SPX domain acts as a sensor in PSR. This list of genes also consisted of purple acid phosphatases, transporters, SQD and DGDG2. Out of the 935 genes, the promoters (2 kb) of 448 (~ 48%) of them had at least one P1BS site. Interestingly, Solyc01g091870 and Solyc05g010060 gene promoters lacked a P1BS site while all the other five SPX domain containing genes (Solyc08g060920 [4 sites], Solyc12g009480 [2 sites], Solyc01g090890 [2 sites], Solyc02g067160 [3 sites] and Solyc02g088210 [3 sites]) contained at least 2 P1BS in their promoters.

**Fig. 6:**
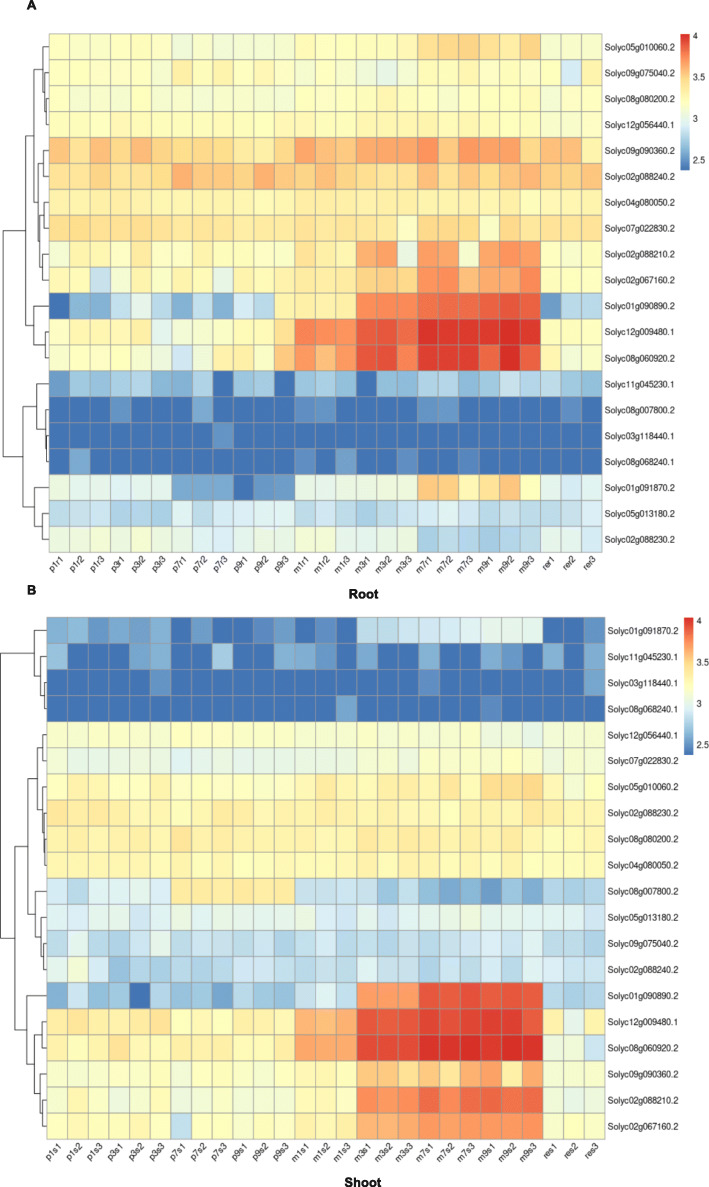
SPX domain-containing genes in tomato are differentially expressed under Pi starvation. The expression profile of 20 SPX domain-containing proteins in root (**A**) and shoot (**B**) samples, respectively. Homologs of AtSPX1, AtSPX2, AtSPX3, AtSPX4 in tomato show strong upregulation under Pi starvation in both root and shoot samples

We then identified the hub genes in the red module. Hub genes are those that have strong connections within a given module and are considered possible regulators of that module (Mason et al. 2009). Among the top 10 hub genes identified, Solyc05g014550 is a TRAM/LAG1/CLN8 (TLC) lipid-sensing domain. Solyc02g089840 (TF protein of unknown function DUF607), Solyc04g080480 (AMP-dependent synthetase and ligase), Solyc06g031710 (nodulin MtN21/EamA-like transporter family protein), Solyc02g031840 (potassium transporter), Solyc11g010700 (receptor-like protein kinase), Solyc12g006130 (F-box family protein), Solyc08g080150 (TCP family transcription factor), Solyc10g017580 (Digalactosyldiacylglycerol synthase 2 [DGD2]), and Solyc06g034290 (glycerol-3-phosphate transporter). The role of Arabidopsis DGD2 is well-established under Pi starvation (Ge et al. 2011). The Arabidopsis homologue of Solyc06g034290 is AtG3PP1, which belongs to the Pi starvation-induced glycerol-3-phosphate permease gene family and its role in Pi ion homeostasis is reported (Müller et al. 2007). All these genes showed strong connectivity (> 0.98) to the red module. Of the 10 hub genes identified, four viz., Solyc04g080480, Solyc05g014550, Solyc11g010700 and Solyc12g006130 have the P1BS in their promoters and could be direct targets of the PHR1 transcription factor.

The blue (3042 genes) and brown (2554 genes) modules show the genes with expression at basal levels 1 and 3 DAT and highly upregulated 7 and 9 DAT. The GO enrichment for the blue module contained the following terms, “water soluble vitamin metabolic process”, “kinase activity”, “protein phosphorylation” and “transferase activity”. In the brown module the terms “RNA metabolic process” and “senescence” were enriched. After resupply, eigengene expression in the blue and brown modules falls to levels similar to the eigengene level under Pi-replete condition at 9 d. These genes are those that are activated on long-term starvation. In the cyan module (281 genes), the eigengene expression is the highest at 1 and 3 DAT, and reduces at 7 and 9 d. A few enriched GO terms are “response to nitric oxide”, “lipid biosynthetic process” and “cellular response to reactive oxygen species”. The eigengene expression is reduced below basal level after resupply. In the greenyellow module (454 genes), the eigengene expression reduces in the first and third days, increases significantly at 7 d and falls at 9 d. After resupply the expression of the eigengene is reduced to near basal level. Some top GO terms enriched in this module are “magnesium ion transport”, “RNA interference”, and “response to wounding”. In the pink module (595 genes), the eigengene expression is activated after 7 and 9 d under Pi-replete condition. Under Pi-depleted condition, it is deregulated significantly at 7 and 9 d. The most enriched term in this module is “response to wounding” and other enriched terms are “response to biotic stimulus” and “immune system process”. It has been previously shown that Pi starvation can lead to suppression of the immune system which results in enhanced plant-microbe interactions (Castrillo et al. 2017). Only a few modules have been described based on responses launched by the plant system during Pi starvation. The genes in the red, blue and brown modules from the root, and in the brown module from shoot are provided in Supplementary Data 2.

The same set of 26,350 genes were used in the identification of co-expression modules in shoot. In all, 41 colour-coded modules were identified of which 12 are shown in Fig. 4. The brown module (4742 genes) consists of an eigengene that largely increases from the 3 d onwards and the expression at 7 and 9 d is similar. The SPX domain-containing genes observed in the red module of the root samples, were also observed in this module in the shoot (Fig. 6B). The expression drops to basal level after resupply. Therefore, this module consists of genes that are highly are highly inducible to Pi starvation. Enriched GO terms included “response to chitin”, “cellular response to starvation”, “phosphorus metabolic process”, and “phosphorylation”. Between the genes in the brown module and red module of the root samples, 471 genes are common. Analysis of promoters of the brown module genes showed that ~ 43% contained the P1BS. The turquoise (7883 genes) and yellow modules (1895 genes) are deregulated on Pi starvation and restored to basal levels after resupply. GO terms enriched in the turquoise module are “photosynthesis”, “cellular component organization and or biogenesis”, “cell cycle” and “developmental process”, and “peptide biosynthetic process”, “gene expression”, and “RNA processing” in the yellow module. Compared with the root samples, the number of shoot modules that show a clear indication to Pi starvation-related modules are fewer possibly due to far more distinct roles played by PSI genes in the root.

### Gene expression changes on resupply of Pi to starved seedlings

After 7 d of subjecting the plants to Pi starvation, a set of plants were resupplied with Pi. DEG analysis revealed the upregulation of 1788 and 2099 genes (log2FC > 1) and 2482 and 2989 genes were downregulated (log2FC < − 1) in root and shoot, respectively. In the root, of all the genes that were upregulated at 9 d between Pi-starved and Pi-replete conditions (log2FC > 1) only three (Solyc10g079320.1, Solyc02g085480.2, Solyc12g096310.1) were observed to be upregulated between Pi-starved and following resupply, while 974 were downregulated. GO analysis of the downregulated genes showed that most of the terms that were enriched under Pi starvation such as “response to starvation”, “transmembrane transport”, “cellular lipid metabolic process” among others, were enriched. The expression level of the rest of the genes (811) were considered to be deregulated to their basal levels. Some of the GO terms that were enriched among these genes were those that were related to “redox homeostasis” and “transcription factor activity”. On the other hand, while 73 genes stayed downregulated in resupply condition, 1153 genes were upregulated (log2FC > 1) in the genes that were downregulated between Pi-starved and Pi-replete conditions. The upregulated genes were enriched for terms such as “cell cycle” and “DNA replication” and other terms that were related to the functioning of the cell machinery. The expression of 1256 genes were reduced to their basal levels. The most enriched term in this set of genes was “response to stimulus”. A similar analysis in the shoot revealed that there were four genes that remained upregulated (Solyc11g066390.1, Solyc09g005400.2, Solyc04g011870.1 and Solyc08g074620.1), 2082 were downregulated and 862 genes were brought back to their basal levels of expression. The genes that were downregulated were enriched for terms such as “response to stress”, “defense response” and “hydrogen peroxide metabolic process”, while some of the GO terms that were enriched for the genes that returned to basal level of expression were “oxidation-reduction process”, “defense response” and “response to stress”. Among the downregulated genes between the Pi-depleted and Pi-replete conditions in shoot, 20 remain downregulated in the resupply plants, while 1304 and 716 genes were either upregulated or restored back to their basal levels of expression, respectively. The most enriched term for the upregulated genes was “translation” and that for the genes that returned to basal level of expression was “glucuronate metabolic process” and “oxoacid metabolic process”.

### Molecular responses of genes involved in ROS production and scavenging, MAP kinases and genes of the anthocyanin biosynthetic pathway

**Fig. 7:**
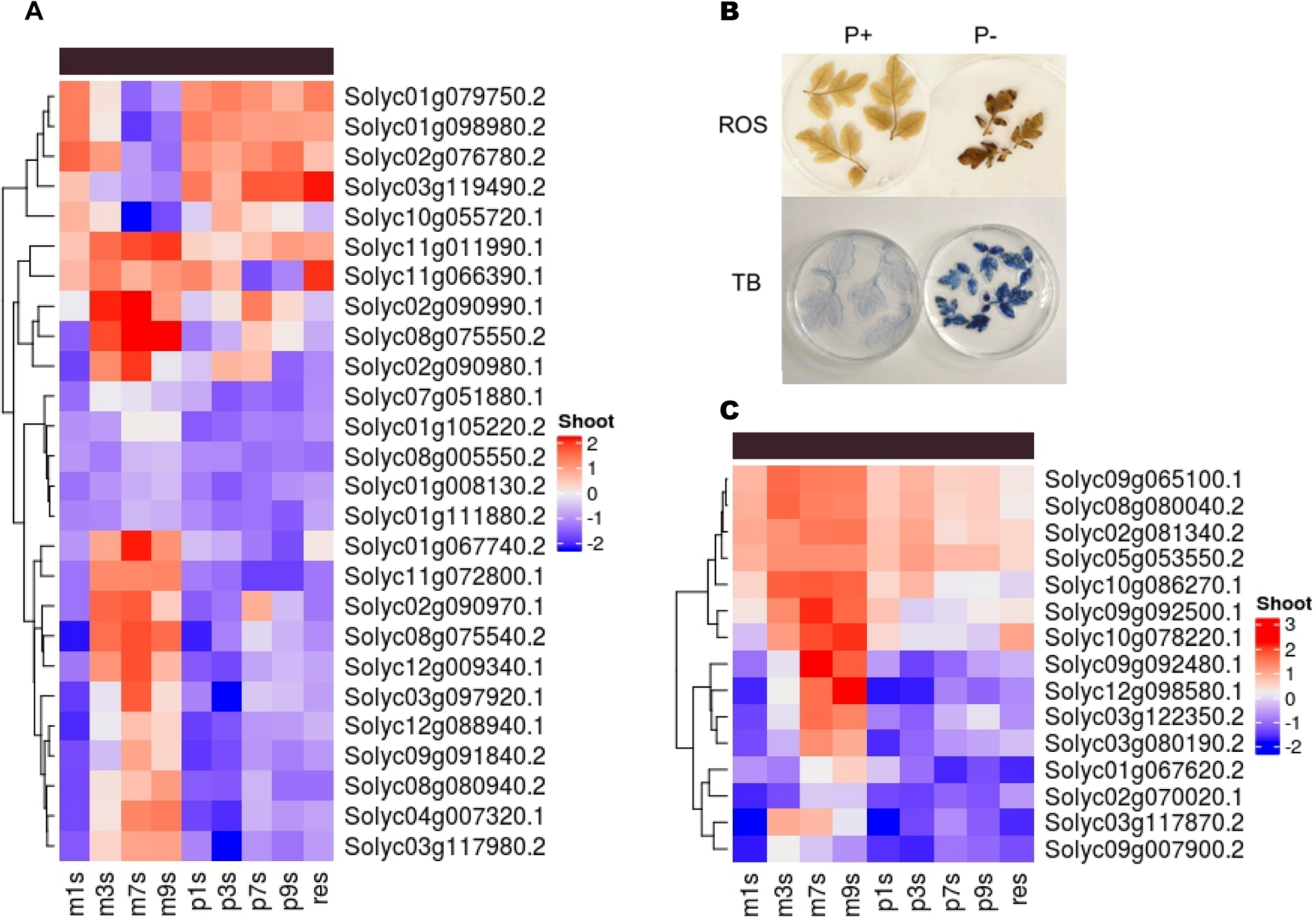
Genes of ROS, MAP kinase and anthocyanin biosynthesis pathway genes are differentially expressed. In shoot, across the timepoints, profile of differentially expressed ROS and MAPK genes (**A**) and anthocyanin biosynthetic genes (**C**). The physiological symptoms of ROS accumulation and cell death observed in leaves 7 d under Pi starvation (**B**). Experiments in (**B**) were performed three times

A total of 91 and 123 genes involved in ROS production and scavenging and MAP kinases, respectively, were identified from the tomato genome. The number of ROS related genes and MAP kinases that were reported to be differentially expressed by DESeq2 analysis is 45 and 41, respectively, in 7 d shoot samples. Of these, 25 and 14 ROS and MAP kinase genes, respectively, were expressed at levels higher or lower than two-fold. The number of genes that were upregulated more than two-fold was 12/9 (ROS/MAP kinases). Six ROS-related genes viz., Solyc03g117980.2, Solyc08g080940.2, Solyc11g072800.1, Solyc08g075540.2, Solyc01g067740.2 and Solyc09g091840.2, and five MAP kinase genes, Solyc12g088940.1, Solyc04g007320.1, Solyc12g009340.1, Solyc03g097920.1 and Solyc02g090970.1 are upregulated in shoot particularly at 3, 7 and 9 DAT (Fig. 7).

For the production of ROS, the H_2_O_2_ marker was used to test tomato leaves 7 d after treatment (DAT). The leaf samples that were taken from seedlings subjected to Pi starvation displayed brownish coloration while the control samples showed no such accumulation (Fig. 7B). This was a clear indication of the accumulation of ROS in the leaf tissue. Similarly, the extent of cell death was also observed in leaf tissue 7 DAT using trypan blue staining. Blue patches were visible revealing the fact that tissues were undergoing extensive cell death. These tissues also displayed symptoms of necrosis. The anthocyanin content was also measured in leaf tissue from treated and control plants. The increase in anthocyanin content 7 DAT was significant. There was an 8-fold increase in anthocyanin content in the leaf tissue of treated seedling. All these physiological responses were indicating that major changes occur in tomato 7 d of Pi-deficiency.

The number of genes that are related to anthocyanin biosynthesis that were upregulated in 7 d shoot under Pi starvation condition was 15. Among these genes, there were two transcription factors (TFs), one MYB (Solyc10g086270.1) and the other a BHLH TF (Solyc09g065100.1), which are both probable positive regulators of the anthocyanin biosynthetic pathway. Some of the other important genes that were upregulated include ACC oxidase (Solyc01g067620.2), glutathione S-transferase (Solyc02g081340.2), 4-coumarate CoA ligase (Solyc03g117870.2), chalcone synthase (Solyc05g053550.2) and anthocyanidin synthase (Solyc08g080040.2). The MYB TF, Solyc10g086270.1, was upregulated in 3, 7 and 9 d Pi-starved shoot. Other genes that showed higher expression levels in these three time points were a UDP-glucuronosyltransferase family 1 protein (Solyc09g092500.1) and a cytochrome P450 (Solyc10g078220.1). The higher expression levels of several of these anthocyanin biosynthetic pathway genes corroborates with the increase in the anthocyanin content in the shoot.

### A subset of genes is alternatively spliced across root and shoot tissues

**Fig. 8:**
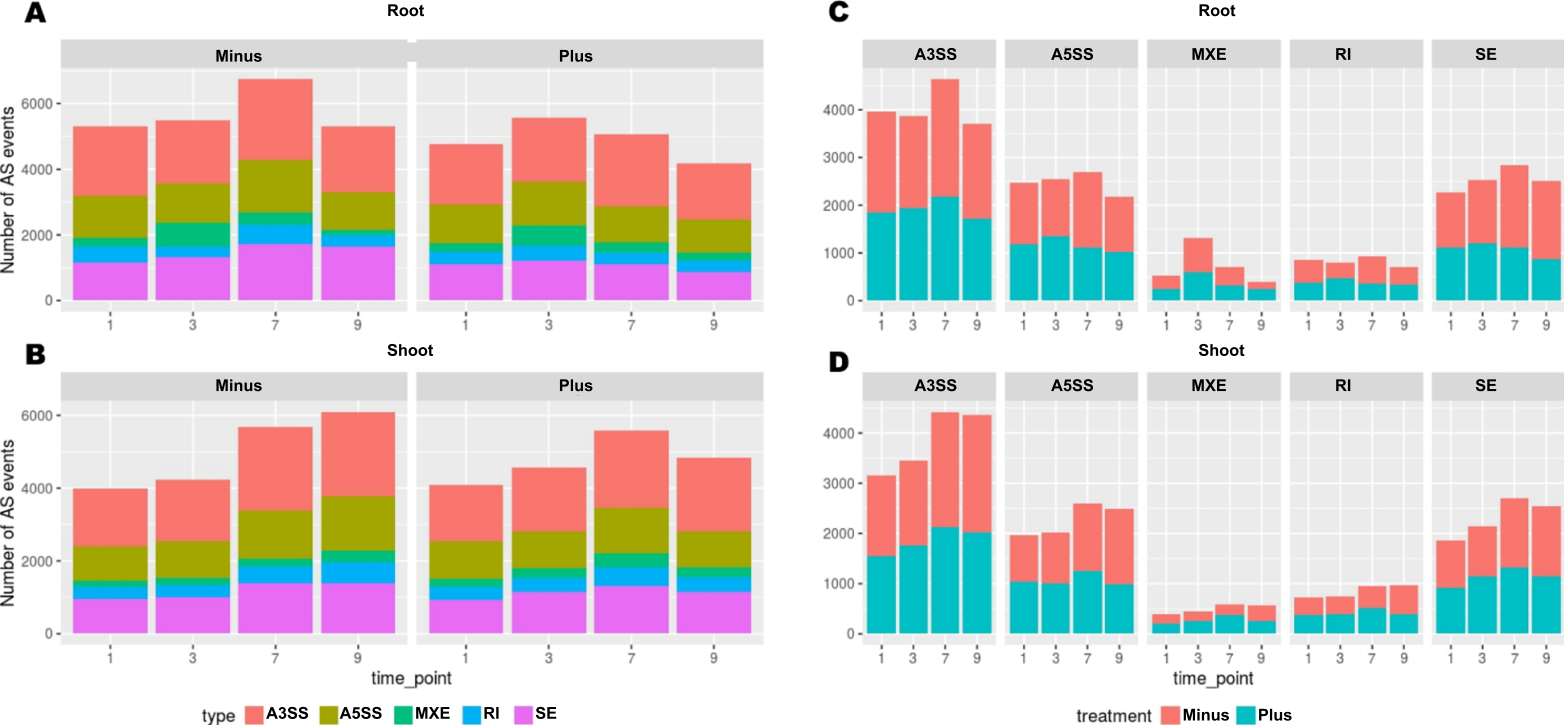
Extensive alternative splicing observed under Pi-depleted condition. The number of different types of alternative splicing events and total number of events under Pi-depleted and Pi-replete conditions in root (**A** and **C**) and shoot (**B** and **D**). Minus – Pi-depleted condition. Plus – Pi-replete condition. A3SS – 3′ alternative splice site. A5SS – 5′ alternative splice site. MXE – Mutually exclusive event. RI – Retained intron. SE – Skipped exon

The alternative splicing landscape was also analyzed, particularly differential alternative splicing (DAS). This was done with rMATS and estimated five different AS events such as exon skipping, mutually exclusive exons, alternative acceptor, alternative donor and retained introns. The number of significant AS events (FDR < 0.05) is only marginally higher under Pi deficient condition than sufficient condition in both root and shoot tissue. The total number of AS events that were identified from 9128 genes was 81,500 from both root and shoot from an overall total of 25,658 intron containing genes (Fig. 8; Supplementary Data 3). Of these, the highest number of AS events were A3SS, followed by SE, A5SS, RI and MXE. While 7484 genes were differentially alternatively spliced (DAS) in the root in all timepoints, in shoot there were 7423. In root tissue, under Pi-starved condition the number of AS events slightly increased 7 DAT and dropped at 9 d (Fig. 8A). The number of mutually exclusive exon events in root, irrespective of treatment condition, decreased considerably in 7 and 9 d (Fig. 8C). A similar trend was also observed in the shoot except that for MXE events, there was a spike in 3 d root. For further downstream analysis, one more step of filtering was introduced. All AS genes that had an inclusion level difference ≥ 0.1 or ≤ − 0.1 were retained. This reduced the number of DAS genes to 6899 with a total of 5118 and 5309 genes in root and shoot, respectively. This is 26.9% of the intron-containing genes in the tomato genome. There were 3528 genes in common that were alternatively spliced in root and shoot. Enrichment analysis showed that GO terms such as “DNA repair”, “intracellular transport”, “DNA metabolic process”, “cellular response to stress” and “RNA processing” were amongst the most enriched. There were also 1590 and 1781 genes that were specific to root and shoot, respectively. The GO enrichment for genes that have higher inclusion levels under Pi starvation and Pi sufficient conditions was also studied. In root, considering all time points, there were 9257 AS events from 3776 genes that had higher inclusion levels under Pi-deficient conditions.

### Differentially expressed and differentially alternatively spliced genes have minimal overlap under low Pi stress

All the genes that were differentially expressed in the root and shoot tissues were identified (log2FC > = 1; adjusted *P* value < 0.05). The number of genes that were upregulated in root and shoot was 3253 and 4789, respectively, and the number of genes downregulated was 3982 and 4620, respectively. There was a total of 6942 and 7795 genes differentially expressed in root and shoot, respectively. Taking into consideration both root and shoot tissues, 12,145 out of 35,358 (34.35%) genes were differentially expressed during the period of treatment. When comparing the DEGs with the DAS genes in root tissue, 1145 genes were identified in both data sets. A GO ontology analysis of these overlapping genes revealed that the most enriched terms were related to DNA repair. Some of the other enriched terms included those related to the lipid metabolic process, “response to stress”, “response to starvation” and “cellular response to stimulus”. These terms indicate that differences in the response to stress not only arises due to changes in differences in expression but also due to alternative splicing. A similar analysis with DEGs and DAS genes from the shoot tissue was also indicative of this fact. There was an overlap of 1346 genes and in this set of genes “organonitrogen compound metabolic process” was the most enriched. Interestingly, apart from the “lipid metabolic/biosynthetic process”, the GO term “superoxide metabolic process” was also enriched. So, here again it is clear that genes that are involved in stress response, are not only differentially expressed but also differentially alternatively spliced. Once the common genes that are both DE and DAS were analyzed for their role in low Pi stress related processes, the genes that were common among these common sets of genes from root and shoot tissue were also analyzed. There were 364 genes that were both DE and DAS that were found to be common in both root and shoot of tomato under Pi starvation.

The top two most-enriched GO terms among this set of genes were “cellular response to stress” and “cellular response to DNA damage stimulus”. The overlap between the DEGs and DASGs is low. Except for a few processes that are common between DEGs and DASGs, there a many other processes that work in concert both through DE of genes as well as DAS, which emphasizes the fact that studying gene expression profiles alone would not suffice in understanding Pi starvation.

### Isoform switch analysis

**Fig. 9:**
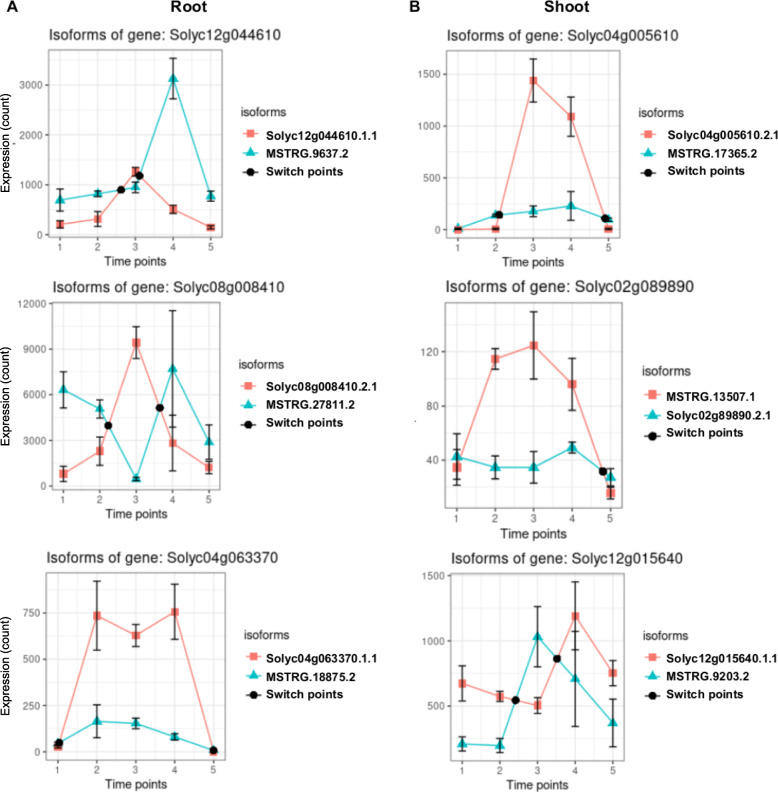
Alternatively spliced transcripts show isoform switching. Isoform switching shown in several genes in root (**A**) and shoot (**B**) samples across the timepoints under Pi-depleted condition. 1,2,3,4 refer to samples under Pi-depleted condition at 1, 3, 7, and 9 days after starvation. 5 refers to Pi resupply

Among the ROS-related genes, 25 and 31 in root and shoot, respectively, were DAS and 12 genes were specific to shoot. Similarly, among the MAP kinase genes, 47 and 40 in root and shoot, respectively, were DAS and 9 were specific to shoot. GO enrichment revealed that 21 ROS-related genes and 22 MAP kinase genes were enriched for “response to stress” and “response to stimulus”, respectively. AS eventually leads to the production of several different isoforms, which could be used differentially in plants under different conditions, a process known as isoform switching (IS), and the isoforms by their inherent difference in functional potential can have a major biological impact (Vitting-Seerup and Sandelin, 2017). An isoform switch analysis was therefore performed for PSR, ROS, MAP kinase and anthocyanin genes that were found to be alternatively spliced. For differential usage of isoforms, the time point at which there is a switch in the isoforms that are preferentially used by the plants need to be identified. For this purpose, the TSIS package in R (Guo et al. 2017)—a program used for the identification of ISs in time-series data—was used to identify the switches in both root and shoot under Pi-depleted and Pi-replete conditions. The genes that are grouped under the red module in the root samples from the WGCNA analysis, and those from the brown module in the shoot samples, were used for this analysis. Several genes showed isoform switching under Pi-depleted condition in both root and shoot. The genes Solyc12g044610, Solyc08g008410 and Solyc04g063370 are shown as examples from the root samples, while Solyc04g005610, Solyc02g089890 and Solyc12g015640 are shown from the shoot samples (Fig. 9; supplementary data 3). Solyc04g005610 showed IS in both root and shoot samples, albeit at different timepoints (data not shown), while the Solyc02g089890 and Solyc12g015640 did not show IS in the root samples. Therefore, IS maybe both condition- and/or tissue-specific.

Amongst the ROS-related genes, Solyc06g068680 exhibited IS with three of its isoforms. When queried against the *Arabidopsis thaliana* proteins, the top hit was RBOHD (AT5g47910), which has been implicated in oxidation-reduction process including negative regulation of programmed cell death and iron-deficiency stress (Zhai et al. 2018, Xie et al. 2011). IS analysis of these isoforms was performed in the shoot samples that were obtained from Pi-replete condition and no IS of these transcripts was detected. A similar analysis performed for MAP kinases identified isoforms from Solyc02g093410, a tyrosine-protein kinase, as showing IS in Pi-deficient shoot. These two genes, Solyc06g068680 and Solyc02g093410, though they were subject to AS, were not DAS. However, they show an additional level of regulation at the level of IS under low Pi condition, which is a likely response to Pi starvation (supplementary Fig. 1; supplementary data 3. Therefore, several layers of regulation seem to operate in the adaptation of a plant to its environment.

## Discussion

Consequential responses of plants *vis-a-vis* environmental perturbations, particularly to nutrient deficiencies, has been much studied in plant biology. Next to Arabidopsis, rice is the most studied plant system for PSR. Recently, two large-scale studies were performed to understand the transcriptomic changes in rice under Pi starvation using RNA sequencing (Secco et al. 2013; Dong et al. 2018). The most elaborate transcriptome analysis of the Arabidopsis response to Pi starvation was a study conducted more than a decade ago using microarray technology (Misson et al. 2005). Most studies have concentrated on understanding gene level changes in the transcriptome during Pi starvation and it has been shown recently that apart from differential expression of transcripts, AS also plays a role in mediating responses under stresses such as Pi deficiency (Dong et al. 2018) and cold (Calixto et al. 2018). In this study, a comprehensive overview of the transcriptomic changes in tomato under phosphate deficient condition is illustrated by analyzing both differential expression and differential AS of genes.

Physiological responses such as Pi content, anthocyanin content, ROS accumulation and cell death made it obvious that major changes were occurring in the tomato plants under Pi starvation (Fig. 1&7). This was also vividly observed with the gene expression changes as there was a dramatic increase in the number of genes that were differentially expressed 7 DAT (Fig. 2). A previous study (Secco et al. 2013) showed that in rice major changes occurred 21 DAT including changes in the expression. This is a clear difference between tomato and rice in their responses to Pi starvation. Another major change that could be observed was the ~ 8-fold increase in anthocyanin content in shoot tissue of Pi-starved seedlings (7 d). Several genes involved in the ROS signaling pathway and MAP kinase genes also were upregulated in shoot tissue 7 DAT which also correlated with the higher accumulation of ROS and increased incidence of cell death in leaf. The genetic interaction between *PDR2–LPR1* mediates accumulation of Fe in root, which in turn activates ROS generation (Müller et al., 2015; Hoehenwarter et al. 2016). Apart from the increase in expression of ROS related genes in shoot, there were also quite a few genes that were upregulated in root tissue and functional enrichment analysis (GO analysis) revealed that “response to reactive oxygen species” was one of the most enriched (*p* value < 0.01) terms in both root and shoot tissue. Although ROS accumulation is a consequence of several environmental stresses, the final response is fundamentally different and the activation of MAP kinases could be through multiple ROS pathways (Pitzschke and Hirt, 2006). Apart from being DE, several ROS-related genes and MAP kinases were also DAS in the present study. Therefore, there are several layers of regulation acting on these genes. It has also been shown that ROS could be a potential signal in mediating Pi starvation responses in the root, particularly, and in the shoot as well (Tyburski et al. 2009; Puga et al. 2017). Of the different ROS, hydrogen peroxide (H_2_O_2_) is the only species that can traverse the plant membranes and therefore has the potential to function as a signaling molecule (Apel and Hirt, 2004).

Many different MAPKs cascades can be activated following ROS accumulation. These include the ROS-responsive MAPKKK MEKK1, MPK4 and MPK6 (Jammes et al. 2009; Xing et al. 2008, Teige et al. 2004). MAP kinases play significant roles in several signaling networks and have been implicated in nutrient signaling, including phosphate starvation (Chardin et al. 2017). In Arabidopsis, *MPK3* and *MPK6* expression is upregulated and MPK3 and MPK6 are activated in response to low phosphate concentrations. Arabidopsis plants impaired in either *MPK3* or *MPK6* take up less phosphate whereas plants in which MKK9, one of the upstream activators of MPK3/6, is constitutively activated, have enhanced phosphate uptake and upregulation of genes involved in phosphate-acquisition. The genes coding for transcription factors HRS1 and WRKY75, which are involved in the interplay between nitrate and phosphate signaling, are regulated by this MAPK module. With several of the MAP kinases activated, similar mechanisms could be operational in tomato too. A closer look at the anthocyanin biosynthetic pathway genes that were DE also showed that the changes in anthocyanin content in the leaf tissue could be attributed to the increase in expression levels of these genes. It is also interesting to note that, UDP-glucosyltransferase (UGT) (Solyc02g070020.1), one of the upregulated genes under Pi starvation, was also found to be one of the driver genes in the pink module. Ectopic expression of UGTs have been shown to increase anthocyanin accumulation and they have also been implicated in enhancing antioxidant activity thereby helping plants to cope with abiotic stresses (Li et al. 2017). Glutathione S-transferase (GST) (*Solyc02g081340.2*), another gene upregulated in the shoot under Pi starvation and it has been reported that GSTs are required for the transport of anthocyanin (Marrs et al. 1995; Mueller et al. 2000). The presence of these genes at higher levels of expression indicate that they might play important roles in the accumulation of anthocyanin and it also could modulate ROS activity during Pi starvation stress (Fig. 7). Therefore, 7 d post treatment in tomato seedlings is an important stage of the plant to study molecular mechanisms that are activated in response to Pi starvation. While major gene expression changes occur at 7 d of Pi-deficient condition both in root and shoot, several important changes occur in root at 3 d (Fig. 2). One of the most enriched terms in the functional annotation of the upregulated genes at 3 d is “cellular response to phosphate starvation”. Therefore, several key genes which are specific to Pi starvation are upregulated early and in fact there are at least 97 of these that stay upregulated up to 9 d of treatment in root. Therefore, several major changes occur between 3 and 7 d and it would be interesting to study pathways such as the lipid metabolic process that are specifically activated during this period.

Originally developed for microarray data, weighted correlation network analysis (WGCN>A) is being used increasingly for the creation of correlation networks for gene expression data generated by RNA sequencing. WGCNA can be used for the identification of modules of genes with high correlation and for eventually the summarization of such modules with the help of the module eigengene or the intramodular hub gene (Langfelder and Horvath, 2008). A total of 26,350 genes were subject to WGCNA analysis. The analysis led to the identification of several distinct modules in both root and shoot tissue samples (Figs. 3-4). In root, the red module with 935 genes contained the Pi starvation-inducible genes such as the SPX-domain containing genes, transporters and purple acid phosphatases and this module therefore is more specific to Pi starvation as the genes returned to basal expression levels after resupply. In shoot samples, the most of the Pi starvation-responsive genes were found in the brown module with 4742 genes. A recent study in tomato cultivar MicroTom identified genes that are DE and DAS under Pi starvation, and further revealed that the cytosines of these genes were not differentially methylated and were abundant in non-expressed genes (Tian et al. 2021). Another study, also in MicroTom, SlPHL1 complements *AtPHR1* mutation and could bind to the P1BS motif and was shown to interact with the promoters of *SlPht1;2* and *SlPht1;8* (Zhang et al. 2021). Interestingly, in our analysis we found that *SlPHL1* gene expression was slightly increased and is part of the red module in the root samples. *SlPHR1/2* were found in the brown module. The coiled-coil domain of PHR transcription factors is important for the interaction with SPX domains and SlPHL1 containing a MYB and coiled-coil domain is a transcription factor localized to the nucleus and could play a significant role in driving the PSR response in tomato (Zhang et al. 2021).

During Pi starvation there are dynamic changes occur at the gene and transcript levels. The extensive AS information identified here demonstrates a much higher degree of complexity of regulation under Pi starvation and it would only be significantly underestimated if analysis were at the differential gene expression alone. In particular, the dynamic contribution of AS by Pi deficiency accompanying the transcriptional response has been shown in this study (Fig. 8). There are over 1400 genes in root and over 1300 in shoot that show regulation both at the levels of gene expression and AS and the rest show changes only at the AS level or DE level alone. Therefore, the massive changes in expression and AS involving a couple of thousand genes reflects the activation of both transcription and splicing pathways and networks. The extent of the Pi starvation-induced AS suggests that AS, along with the transcriptional response, is a major driver of transcriptome reprogramming for adaptation to Pi deficiency. Recent studies have shown that AS is involved in ~ 60% of intron-containing genes in Arabidopsis (Marquez et al. 2012; Zhang et al. 2017), 52% in soybean (Shen et al. 2014), ~ 40% in maize and cotton (Thatcher et al., 2016*:* Li et al., 2014) and 53% in rice (Dong et al. 2018). In the present study, for the first time, about 27% AS in tomato is reported only at the DAS level among the intron-containing genes. There are at least 34% of these genes showing AS, a percentage not comparable to that of other model plants (Marquez et al., 2012; Shen et al., 2014; Thatcher et al., 2016; Dong et al. 2018) as the tomato genome is still being actively curated and improved upon, which will help in the identification of several more genes that are AS. A higher proportion, 44% and 25% of the uniquely alignable mRNA-seq reads, was in the intergenic and intronic regions, respectively, as compared to 23% and 15% from the mRNA-seq data set (Cui et al. 2010). Also, further analysis showed that different library construction methods have significant impact on the gene expression profiles. Therefore, in this study, the occurrence of retained introns is not significant when compared to other types of AS events such as A3SS, A5SS, and SE.

Analysis of the list of genes with DASGs under Pi deficiency reveals that several Pi-related genes that play roles in transcriptional and post-translational regulation are alternatively spliced during Pi starvation, including the SPX domain-containing proteins. This finding suggests that AS has an important role in regulating P-deficiency responses and Pi uptake and distribution in plants. During Pi starvation, there are alterations in the membrane lipid composition in plants, and enzymes such as phospholipase D and phosphatidate phosphatase are involved in the degradation of phospholipids releasing diacylglycerols that are used in the glycolipid synthesis. These genes along with the digalactosyl diacylglycerol synthase 2 (DGDG) and sulfoquinovosyl transferase genes, involved in lipid biosynthesis pathway, and the *Tomato Phosphate Starvation Induced 1 (TPSI1),* one of the first genes characterized in tomato under Pi deficiency condition (Liu et al. 1997, Yu et al. 2002; Siebers et al. 2015), were also upregulated in all time points. The activation of these genes clearly revealed that most of the pathways in plants that are affected by Pi starvation are similar and studies in model organisms such as Arabidopsis help us in understanding these pathways in non-model species with more accuracy taking into the subtle differences that occur between species.

In addition to DEGs, genes that are DAS too are an integral part of the stress-response mechanism of a plant. This is consistent with a recent study showing that changes in transcript structure contribute to the transcriptome complexity in Arabidopsis plants under nutrient stress (Nishida et al., 2017). Comparative analysis of DEGs and DAS genes under nutrient deficiency conditions revealed that there was little overlap between them, and similarly between functional groups (GO analysis). A similar finding was reported in two AS studies of Arabidopsis (Li et al., 2013; Nishida et al., 2017; Dong et al. 2018; Calixto et al. 2018). Interestingly, GO enrichment analysis of the DAS genes in the current study included nucleic-acid metabolic pathways, mRNA splicing, and protein kinases that regulate gene/protein expression and function, similar to the situation in Arabidopsis (Nishida et al., 2017; Dong et al. 2018). The regulation of transcription and mRNA splicing, therefore, is controlled by an independent regulatory system that is conserved among different plant species and supports the hypothesis that environmental and developmental cues affect gene expression at the level of transcription and AS, which are mediated by transcription factors and splicing factors, respectively (Staiger and Brown 2013).

The AS of several genes implicated in the P-signaling pathway in rice such as PHO2, PHR homologues and SPX-domain containing proteins has been reported previously (Secco et al. 2013; Dong et al. 2018). Many putative homologues of the Arabidopsis Pi-signaling genes in tomato displayed DAS both in root and shoot and of particular interest is the AtSIZ1 homologue, Solyc11g069160.1. While it was not DE, it was DAS under Pi starvation (data not shown). AtSIZ1 is a (Sumo) E3 ligase that SUMOylates AtPHR1 and activates it during Pi starvation (Miura et al. 2005). Therefore, a focal regulator of PSR being regulated at the level of mRNA splicing warrants further study. This also reiterates the fact that AS plays a major role in PSR.

The PSR pathway in tomato probably involves the anthocyanin, ROS and MAP kinase signaling cascades that affect the expression and AS of specific genes in the respective pathways. The Pi starvation-induced changes in expression and AS suggested a model where Pi sensing and signaling pathways modulate both transcription and splicing activity. The changes noted in several of these different genes determine the overall reprogramming of the transcriptome for adaptation to low Pi stress. The DE and DAS gene sets that have been observed across the various time points are testimony to the fact that these cascades are dynamically affected.

## Conclusion

Plants, being sessile, are forced to adapt to varying environmental conditions wherever they grow, which necessitates the presence of complex mechanisms to cope with these changes. They require flexible regulatory systems that modify expression quickly and reversibly upon perception of constantly fluctuating nutrient-levels. They must also re-program the transcriptome under Pi deficient conditions to allow the plant to acclimate, use Pi efficiently and survive as the duration of the stress increases. In this study, therefore, a comprehensive analysis of the transcriptome re-programming that is instantiated in tomato on the wake of Pi starvation is presented. Changes both at the gene and transcript levels lead to activation of pathways that then stage their efforts to adapt to the changing environment. As this is the first major study providing an overall view of the transcriptome landscape of tomato under Pi starvation, it would serve as an invaluable resource for all researchers working on tomato and its adaptation to Pi deficiency.

## Methods

### Plant materials and growth conditions

The tomato (*Solanum lycopersicum*) accession Heinz1706 was used in this experiment. The seeds were disinfected with 20% bleach followed by thorough washing five times with distilled water before germination. The seeds were pre-germinated for 1 week before transferring to hydroponic solution containing: 2.5 mM Ca (NO_3_)_2_.4H_2_O, 1.25 mM K_2_SO_4_, 1 mM MgSO_4_.7H_2_O, 12.5 μM H_3_BO_3_, 2.25 μM MnSO_4_.H_2_O, 1.9 ZnSO_4_.7H_2_O, 0.25 μM CuSO_4_.5H_2_O, 0.1 μM Na_2_MoO_4_.2H_2_O, 0.13 μM KCl and (Fe-stock) 8 μM Fe (III)-EDTA with 250 μM KH_2_PO_4_ for Pi-sufficient condition and with 125 μM K_2_SO_4_ for Pi-deficient condition (i.e. 0 Pi). The germinated seeds were transferred to hydroponic medium with 250 μM Pi for 1 day. The following day, half of the seedlings were transferred to a medium with no Pi (0 μM Pi) for a period of 7 days. After 7 d, half of the seedlings from the Pi-deficient medium was transferred into medium containing 250 μM KH_2_PO_4_ for 2 d. Throughout the hydroponic experiment, the pH of the medium was adjusted to 5.7 and was renewed once every 3 days. The plants were grown in a growth chamber under controlled conditions at a temperature of 28/19 °C (16/8 h) day/night cycle and a relative humidity of 60–65%. Root and shoot tissues were collected from the plants separately 1, 3, 7 and 9 d after treatment. Samples from the resupply experiment were collected 2 d after treatment (Fig. 1A). For each sample, 3–4 seedlings were pooled to comprise a single replicate with a total of three biological replicates per treatment. Totally, 54 samples were generated.

### Pi and anthocyanin content estimation

Root and shoot samples were frozen after taking the fresh weight. To the frozen sample, 1% glacial acetic acid was added and mixed thoroughly by vortexing. By repeated freeze-thaw cycles, the contents of the tissues were released into solution. The supernatant obtained after centrifugation was estimated for Pi content using the phosphomolybdate colorimetric assay (Ames, 1966). Extraction of anthocyanin was performed by incubating the leaves in 300 μL of extraction solution (methanol in 1% HCl) overnight at 4 °C. After the extraction, 200 μL each of water and chloroform was added and centrifuged to remove the plant debris. The anthocyanin content was read at A_530_. All the measurements were recorded in eight individual seedlings.

### H_2_O_2_ visualization and trypan blue staining

For hydrogen peroxide staining, leaves were vacuum-infiltrated with 0.1 mg mL − 1 3,3′-diaminobenzidine (DAB, Sigma D12384-1G) in 50 mM Tris-acetate buffer, pH 5.0. Samples were incubated for 24 h at room temperature in the dark before transferring to 80% ethanol. Once chlorophyll was completely removed, the ethanol was replaced with 25% glycerol and the leaves were visually scored for color intensity. For trypan blue staining, the leaf samples were immersed in 0.4% trypan blue solution at room temperature for 8 h. The samples were decolorized by boiling them in a fixing solution (1 lactic acid: 1 glycerol: 4 ethanol) before image documentation.

### Total RNA isolation and mRNA sequencing

From root and shoot of tomato, total RNA was isolated using the RNAprep Pure Plant Kit (Tiangen). To eliminate genomic DNA contamination, DNAse was added during isolation of RNA. The quantity of RNA was ascertained using a NanoDrop 2000c Spectrophotometer (Thermo Fisher Scientific). For mRNA sequencing, three biological replicates were taken and each biological replicate included three plants. The samples were frozen in liquid nitrogen immediately after collection and stored at − 80 deg C until further processing. The NEBNext Ultra II Directional RNA library Prep Kit for Illumina (New England Biolabs) was used for generating the sequencing libraries with a starting material of 4 μg of RNA. Paired-end (150 bp) sequencing was performed using Illumina reagents at the Genomics Core Facility at the Shanghai Center for Plant Stress Biology following the manufacturer’s instructions.

### Read mapping, transcript assembly, quantification and differential gene expression analysis

The raw reads were quality filtered using the bbduk shell script that is provided along with the bbmap suite of tools (version 38.00; https://sourceforge.net/projects/bbmap/files/) with the following parameters: ktrim = r k = 23 mink = 11 hdist = 1 tpe tbo for adapter trimming followed by quality trimming with qtrim = rl trimq = 20 minlen = 25. Clean reads were mapped to the tomato genome build SL2.50 and the associated annotation (ITAG2.4) GTF file obtained from EnsemblPlants (http://plants.ensembl.org/Solanum_lycopersicum/Info/Index) using STAR version 2.5.3a (Dobin et al. 2013) with default parameters. Transcript assembly and quantification was performed with StringTie version 1.3.3b (Pertea et al. 2015). The read count information was extracted with the prepDE.py script provided along with StringTie (http://ccb.jhu.edu/software/stringtie/index.shtml). Only the gene-level read count information thus generated for the genes annotated (ITAG2.4) was used for differential gene expression analysis with DESeq2, an R/Bioconductor package (Love et al. 2014). The differential gene expression analysis between Pi-replete and Pi-depleted conditions for the different samples at each time point for root and shoot samples separately was performed with respect to their corresponding control samples. DEGs displaying two-fold or more changes with an adjusted *P* value <= 0.05 were selected for further analysis. GO enrichment analysis was performed on PlantRegMap (http://plantregmap.cbi.pku.edu.cn/go.php) (Jin et al. 2017). Heatmaps were generated with either pheatmap or ComplexHeatmap, which are both bioconductor packages (https://cran.r-project.org/package=pheatmap; Gu et al. 2016).

### Weighted gene co-expression network analysis

For the unsupervised WGCNA, only genes that have at least for one time point over all three replicates a DESeq2-normalized read count of at least 10 were considered. A total of 26,350 genes passed these filtering criteria. DESeq2-normalised counts then served as input for the network analysis. We used the function blockwiseModules, implemented in the R (www.r-project.org) WGCNA package (v1.51) (Langfelder and Horvath, 2008), to create a signed network of a Pearson correlated matrix. All genes were treated in a single block. The signed network ensured that only positive correlations were considered. A soft power threshold of 7 was chosen because this was the lowest power needed to reach scale-free topology (R^2^ = 0.8). Module detection was performed with default settings (mergeCutHeight of 0.15 and enabled PAMstage). The minimal module size was set to 30 genes. For each module, the expression profile of the module eigengene was calculated, which is defined as the first principal component of the module’s expression data. For each gene, the intramodular connectivity (kME) was calculated, which represents Pearson correlation of the individual gene with the respective module eigengenes.

### Phylogenetic analysis

For the SPX domain-containing genes phylogenetic analysis, candidate SPX domain (domain signature PF03105) sequences were identified from the tomato, rice, Arabidopsis and maize protein sequences downloaded from EnsemblPlants (https://plants.ensembl.org/index.html) using HMMER3.0 with default settings (Finn et al. 2011). The phylogenetic tree reconstruction of these protein sequences was performed with the ETE Toolkit using the “-w standard_fasttree” option with other parameters kept at default values (Huerta-Cepas et al. 2016).

### Alternative splicing and isoform switch analysis

Alternative splicing analysis was performed with rMATS (version 3.2.5) (Shen et al. 2014) with default parameters. The reads were mapped with STAR (version 2.5.3a) in the “--twopassMode” to the tomato genome build SL2.50 and the associated annotation (ITAG2.4) GTF file and the resultant alignment files in the sorted-BAM format were used to run the rMATS program to identify a variety of transcriptome-wide differential splicing events across two conditions. We used sorted-BAM files of samples under Pi-replete as control for each time point and tissue against the Pi-depleted samples, along with replicates as input for the rMATS program and run with default parameters, setting a cutoff at FDR < 0.05. Both junction counts and on-target reads were used to detect significant events. The five different alternative splicing events detected were SE (skipped exon), MXE (mutually exclusive exon), A5SS (alternative 5′ splice site), A3SS (alternative 3′ splice site), and RI (retained intron). The isoform switch analysis was performed using the TSIS program (Guo et al. 2017). For performing this time series isoform switch analysis, the transcript-level count information generated through StringTie was used along with the novel transcripts identified from the annotated ITAG2.4 genes.

### Quantitative real time reverse transcription-PCR

Total RNA was isolated from the tissues using RNeasy Plant Mini Kit (Qiagen, Germany). The cDNA was synthesized from 1 μg of total RNA using the Bio-Rad iScript reverse transcription kit, according to the manufacturer’s instructions. Real time RT-qPCR assay of the transcript levels was performed using the CFX Connect™ Real Time System (Bio-RAD). The *EF1α* gene was used as internal control.

## Supplementary Information

**Additional file 1.**

**Additional file 2.**

**Additional file 3.**

**Additional file 4. Supplementary Fig. 1** Non-differentially expressed, alternatively-spliced genes showing isoform switching. The ROS-related gene Solyc06g068680 (A) and the MAP kinase gene Solyc02g093410 are alternatively-spliced and show IS, but are not differentially expressed.

## Data Availability

The materials used in this study will be available for research upon request.

## Abbreviations

Pi: Phosphate
PSR: Phosphate stress responses
P: Phosphorus
PSI: Phosphate starvation-induced
DEG: Differentially expressed gene
DASG: Differentially alternatively-spliced gene
ROS: Reactive oxygen species
WGCNA: Weighted gene co-expression network analysis
P1BS: PHR1 binding sequenced
TF: Transcription factor
IS: Isoform switch
