# Supplementary material for: High transcriptome plasticity drives phosphate starvation responses in tomato
Source: Stress Biol. 2022 Mar 18;2(1):18. doi: 10.1007/s44154-022-00035-4 (PMC10441952; doi:10.1007/s44154-022-00035-4)
Supplement: Supplementary file 4 — Additional file 4. Supplementary Fig. 1 Non-differentially expressed, alternatively-spliced genes showing isoform switching. The ROS-related gene Solyc06g068680 (A) and the MAP kinase gene Solyc02g093410 are alternatively-spliced and show IS, but are not differentially expressed. [file 44154_2022_35_MOESM4_ESM.pdf]

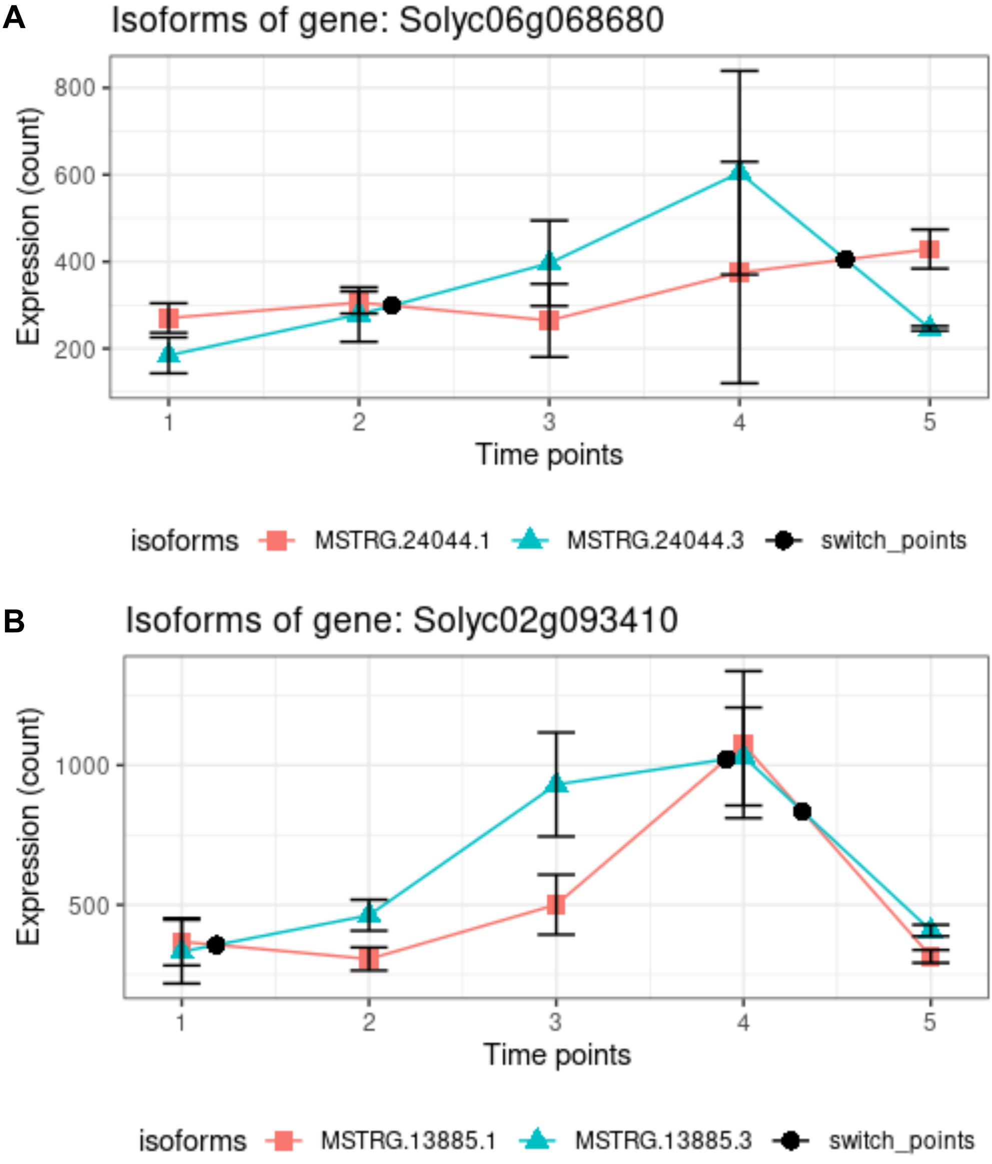
A
Isoforms of gene: Solyc06g068680
Expression (count)
800
600
400
200
1
2
3
4
5
Time points
isoforms
MSTRG.24044.1
MSTRG.24044.3
switch_points
B
Isoforms of gene: Solyc02g093410
Expression (count)
1000
500
1
2
3
4
5
Time points
isoforms
MSTRG.13885.1
MSTRG.13885.3
switch_points
